# Influence of Laboratory Long-Term Aging on Selected Fracture Parameters of Asphalt Mixtures

**DOI:** 10.3390/ma14040811

**Published:** 2021-02-08

**Authors:** Pavla Vacková, Jan Valentin, Majda Belhaj

**Affiliations:** Department of Road Engineering, Faculty of Civil Engineering, Czech Technical University in Prague, 166 29 Prague, Czech Republic; jan.valentin@fsv.cvut.cz (J.V.); majda.belhaj@fsv.cvut.cz (M.B.)

**Keywords:** asphalt mixtures, fracture toughness, SCB test, three-point bending test, fracture energy, aging, aging index

## Abstract

The paper presents the influence of laboratory aging simulation on fracture properties determined on 150 variants of asphalt mixtures. The fracture properties were determined by two different test approaches—semi-circular bending test (SCB test) and three-point bending test on beam specimens (3-PB test). The aging was simulated according to one of the methods defined in EN 12697-52 (storage of test specimens in chamber at temperature of 85 °C for 5 days). The evaluated group of variants covered asphalt mixtures for all road layers. The group was further divided according to used bituminous binder (unmodified vs. modified) and reclaimed asphalt content. The results showed that strength parameters (flexural strength and fracture toughness) increase with aging. It further shows that fracture work provides more complex information about the cracking behavior. For the aging indexes, it was found that for mixtures with modified binders and mixtures which did not contain reclaimed asphalt (RA), the values were higher. The aging indexes for fracture work showed different results for both performed tests.

## 1. Introduction

Aging of asphalt mixture influences all the mixture’s properties. The bituminous binder oxidizes due to exposure to climate condition as temperature, oxygen, ultraviolet light, water, and other external environmental factors, [1,2,3]. It is a natural process which cannot be avoided, it can be only partly controlled. The oxidation causes irreversible (or only very hardly reversible) changes in binder´s characteristics and adversely affects pavement service life. The behavior of asphalt mixture depends mainly on the behavior of bituminous binder [4,5,6,7,8,9].

Bituminous binder is viscoelastic and changes its properties with temperature and time. Bituminous binders consist of four fractions including saturates, aromatics, resins, and asphaltenes [3,10]. The proportion between asphaltenes and maltenes is changing due to aging—aromatics become resins and resins become asphaltenes which leads to increased stiffness, viscosity, and softening point on one hand and decreased ductility and penetration on the other hand. However, the increase of asphaltenes is not the only reason for the aging, the asphaltenes interact with each other and/or with the maltenes and therefore the increase in viscosity also highly depends on the shape of asphaltenes particles [4,11,12,13,14].

It is further known that aging increases resistance to permanent deformation and leads to higher resilient modulus, at the same time it reduces fatigue life [4]. The bitumen aging process also leads to increased brittleness and decreased resistance to sudden changes due to climatic conditions in combination with the repeated effect of traffic loading. The aged binder is less flexible, so it can be easier to initiate and subsequently to propagate cracks, which affects both temperature induced cracks and fatigue cracks. This can eventually cause the service life of pavement to be shortened [15,16].

The low temperature properties are crucial for asphalt mixtures and can significantly influence the service life. Various tests have been developed and applied to evaluate fracture properties of asphalt mixtures such as two/three/four point bending test [17,18,19,20,21], disk-shaped compact tension test (DCT) [22], indirect tensile creep and strength test (IDT) [23,24], semi-circular bending test (SCB) [25,26,27,28], Illinois flexibility index test (FI) [29], etc. These tests differ not only in required equipment and specimen shape, but also in test parameters. Some of the tests use “strength” approaches, when the results are determined focusing on the maximum force and deformation gained within the test run (e.g., fracture toughness, flexural strength etc.). On the other hand, some of the tests use “energy” approach and the results are calculated from force-strain diagrams (J-integral, fracture energy, flexibility index, etc.).

Mohammad et al. (2015) [30] conducted the SCB test to evaluate and compare the performance of laboratory-produced mixtures with the crack pattern obtained from the pavement that has been in service for approximately 10 years. The outcomes showed that cracking rate of the pavement decreased by increment of asphalt mix fracture resistance. Omranian et al. (2018) [8] found that the stiffening of an asphalt mixture by aging increment is beneficial until a certain level based on the higher energy requirement necessary to reach failure, while the detrimental effects of aging are observed once the aging conditioning crosses the fracture resistance level given for an optimal mix. Fakhri et al. (2017) [31] found that laboratory simulation of aging reduces the fracture resistance of asphalt mixes in terms of both critical strain energy release rate (Jc) and flexibility index (FI), however, the statistical comparison shows that the reduction is only significant in case of flexibility index. The results also depend on reclaimed asphalt (RA) content. Saeidi et al. (2016) [32] stated that as the bituminous binder ages, the stiffness increases which leads to consequently fracture energy increase; however, the failure displacement decreases.

The objective of the research study presented by this article is to evaluate different fracture parameters from point of view of asphalt layer service life. The evaluated group of asphalt mixtures covers different kinds of mixtures (asphalt concretes used for different layers, stone mastic asphalt, high modulus asphalt concrete and others). The evaluated group covers also various types of binders (unmodified binders/paving grades as well as polymer or crumb rubber modified binders) and includes even mixtures with elevated content of reclaimed asphalt (RA).

The evaluated data were gathered from different research and commercial project to cover as wide group as possible.

## 2. Materials and Methods

### 2.1. Asphalt Mixture Variants

The comparison of aged fracture parameters was done on almost 100 hot asphalt mix variants for SCB test and almost 150 variants for 3PB test. The results used for evaluation were collected from different studies and commercial tasks. The research data include laboratory produced mixtures as well as mixtures produced by asphalt plants. The used variants differed in aggregate, used type of bitumen as well as source where the bituminous binders originated.

The goal of this research was to compare as wide and diverse a group of asphalt mixtures as possible. All of the variants always fulfilled the valid product standards in terms of voids content, bitumen content, and other required basic parameters. Due to the huge number of assessed mixtures, these basic parameters are not provided in this paper.

As it was mentioned above, the group of assessed mixtures covered all type of asphalt mixtures used regularly in the Czech Republic (asphalt concrete for different pavement layers, stone mastic asphalt, high modulus concrete, noise-reducing asphalt layers, etc.) with various mix designs. The groups of assessed asphalt mixtures were evaluated from two different perspectives—according to used binder (unmodified or modified) and according to presence of RA (virgin asphalt mixtures without any reclaimed material and mixtures with RA; there was no further differentiation if 10% or 50% RA had been used).

The “modified” group included asphalt mixtures with polymer and crumb rubber modified binders. The “RA” group included asphalt mixture with 10 to 60% of RA. The aged binder was restored in case of mixtures with reclaimed asphalt by softer or special binders or by different kinds of rejuvenator. Nevertheless, there was not a deeper subdivision regarding the way how the RA was softened or rejuvenated. Moreover, there was no further division according to the asphalt mixture type, because it was not a task for this article.

### 2.2. Fracture Testing Methods

The fracture parameters were determined by two different methods applied on two different shapes of test specimens. The fracture behavior was assessed for low-temperature range to explain the qualitative potential of frost cracking for tested asphalt mixtures.

The first presented method was semi-circular bending test (SCB test), determined by modified test protocol which was derived from the defined procedures given in EN 12697-44 [33]. The principle of this method is three point bending of semi-circular test specimen with defined notch at the bottom. The modified test protocol uses test specimens compacted according to EN 12697-30 [34] by impact compactor with 100 mm diameter, instead of test specimen with 150 mm diameter compacted by gyratory compactor. The reason for this modification is given by the fact that in most European countries, an impact compactor is a traditional and preferred way of preparing test specimens. The use of gyratory compactor is very limited in many European countries. The modified method uses loading rate of 2.5 mm/min and test temperature of 0 °C. According to the European standard, loading rate of 5.0 mm/min is required. The reason for changing this rate was explained, e.g., in [35,36]. The test parameters are fracture toughness, fracture work/energy till the maximum force, and (total) fracture work/energy. Fracture work is calculated as integral of force-displacement curve and fracture energy is ratio of fracture work and fracture area. (In this article, only fracture work is presented to compare the same parameters for both test methods, even though the fracture energy is a more common parameter for semi-circular bending test).

The second test method was a three-point bending of beam specimens (3PB test) defined by technical specifications TP 151 of the Ministry of Transport of the Czech Republic [37]. The test specimen has length of 300 mm and cross section of 50 × 50 mm^2^, 40 × 50 mm^2^ or 60 × 50 mm^2^ according to maximum aggregate size used in mixture. The loading rate is 1.25 mm/min and the test temperature is the same as for previous test, i.e., 0 °C. The test parameters are flexural strength and fracture work till the maximum force.

The impact of asphalt aging on fracture parameters was simulated according to one of the methods defined in CEN/TS 12697-52 [38]. The specimens are stored in a thermal chamber with air circulation at temperature of 85 °C for 5 days to simulate long-term aging. This method is used over a long period by the laboratory of Czech Technical University. After aging, the specimens were tested at the same conditions as “unaged” (virgin) test specimens.

The aging index determines the influence of laboratory simulation of aging on the properties of asphalt mixture. The aging index is calculated as a ratio of aged and unaged test specimens’ parameters. The closer the aging index is to 1.0 (100%), the less susceptible to aging the test specimen (asphalt mixture) is, and it can be potentially considered as more resistant to cracking ↔ its durability might be improved. Of course, such an assumption is very approximate since there is not a true fatigue loading to the test specimens. It is rather an indication. This hypothesis is based on the fact that the test specimens, which are based on the aging index, less susceptible will demonstrate less brittleness and, therefore, it should resist the cyclic stress in the fatigue test, achieving an improved overall lifespan.

Generally, the strength properties increase (to a certain extent) with aging of bituminous binder. As it is expected during aging, the penetration of bituminous binder decreases, and strength characteristics grow. This applies to flexural strength, where in almost all cases the aging index is bigger than one. The fracture toughness (SCB test) is influenced not only by the strength grow, but more likely by the increase of brittleness. The test specimens have a defined notch on the bottom, which influences the results, and it is not easy to predict the influence of aging on asphalt mixtures. The aging index of fracture toughness depends on many factors as e.g., type of the binder, presence and amount of reclaimed asphalt (RA), used additives, modifiers, and other admixtures.

**Figure 1:**
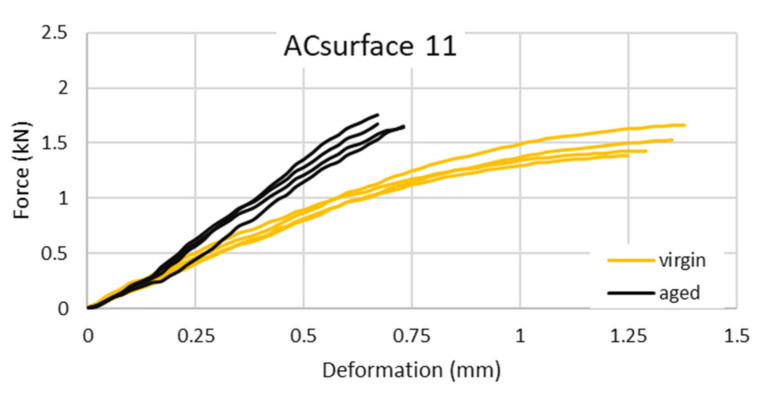
Example of force-displacement diagram for 3-point bending test—AC_surface_ 11 50/70.

In contrast, the fracture work (fracture energy) usually decreases for aged specimens. The aged binder is tougher but less flexible. Therefore, even though mixtures might reach higher maximum force, usually at the same time the deformation is smaller. The typical force-deformation loading diagrams for AC_surface_ 11 with unmodified bituminous binder are displayed in Figure 1 (the typical test set for 3PB test includes 4 unaged and 4 aged specimens).

The impact of aging on asphalt mixtures (asphalt layers in a pavement) is mainly influenced by the aging of bituminous binder, which can be explained as a thermo-oxidative process, i.e., its properties are variable over time due to climatic conditions, atmospheric effects, presence of oxygen, ultraviolet (UV) radiation, etc. [39,40,41]. The pace of aging depends on the binder’s type (unmodified, modified, etc.), on source from which it is produced, the processing technology (distilled, semi-blown etc.), the type and amount of used modifier or additives, storage conditions, etc.

Another factor that significantly affects the aging of asphalt mixtures is presence of reclaimed asphalt (RA), which becomes a more and more common aspect of today’s asphalt mixtures. Although incorporating the reclaimed asphalt into pavement construction is economical and ecologically convenient, the excessive use of RA can without proper design and testing lead to worsened pavement performance and shorter service life. In this case, it is necessary to pay increased attention to proper design and control testing. The fatigue and fracture properties are essential for mixtures with elevated RA content.

In the production of asphalt mixtures with RA, the final product quality depends on the amount of aged binder in RA, type and amount of added virgin bitumen, and also on potential additives (so-called “rejuvenators”), which are used mainly in asphalt mixtures with elevated contents of RA to improve overall properties of asphalt mixtures. The rejuvenator is used to reactivate/rejuvenate the aged binder. Rejuvenators usually affect the penetration or/and softening point of binder and chemically might change the proportion between asphaltenes and maltenes [11,42]. The expected rate of softening can be managed by the type of additive or its dosage. When a larger amount of additive is added, the properties are usually more significantly affected. However, there is risk of worsen resistance to permanent deformation (the mixture can be too soft) and of course the economic point of view plays another important role [43].

The long-term effect of such additives is crucial in monitoring of long-term aging. The additive can only act for a short time, so the properties are mostly improved only during manufacturing and paving, but their effect quickly disappears; or they may have long-term effect, when they functionally improve the properties of the asphalt mixture for many years of operation and a successive diffusion of the acting additive can be observed. Several experimental studies have been performed to determine and describe the diffusion behavior of rejuvenator into aged bitumen. Karlsson and Isacsson (2003) used FTIR-ATR method to study diffusion process [44,45]. It was found that the calculated diffusion coefficient varies depending on the molecular weight and polarity of molecular group observed in rejuvenator-bitumen blend. Ma et al. (2015) measured the distribution of rejuvenator in the recycled asphalt binder using phased extraction and recovery test and found that rejuvenator with lower viscosity had the higher penetration into bituminous binder [30], i.e., the chemical composition and structure of a rejuvenator plays an important role. Guangji Xu et al. (2018) used molecular dynamics to study diffusion of rejuvenators into bitumen. The results of this study indicate that simulations by molecular dynamics have the potential to calculate inter-diffusion coefficient and thermodynamic properties of bituminous binder [7].

## 3. Results

### 3.1. Three Point Bending on Beam Specimens

In flexural tensile test, there is (in almost all cases) an increase in flexural strength and, on the other hand, usually decrease in fracture work.

For more than 90% of variants, due to aging, the flexural tensile strength increased (aging index above 1.0 was reached). The decrease in flexural strength can be caused e.g., by heterogeneity of test specimens, invisible deformation of test beams during the aging, reached limit strength, etc. It might be also caused by averaging of similar strengths as well.

**Figure 2:**
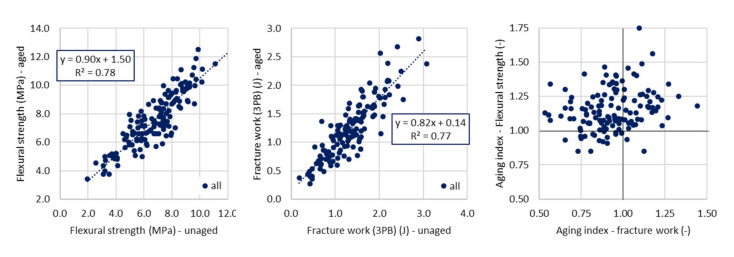
Comparison of aged and virgin parameters—three-point bending test (**left** and **middle**) and comparison of aging indexes (**right**).

The average aging index for fracture work was below 1.0, as expected, but the individual value were “on both sides of 1.0” (Figure 2, right). This fact confirms the stated assumption that due to aging, the stiffness of the asphalt material increases to a certain extent, but at the same time the brittleness increases and thus causes the decrease in resistance to stress in the field of low temperatures and sudden climate changes. Therefore, the aged test specimens need, in low temperature ranges, less energy to be fractured.

There is quite strong correlation between the aged and virgin parameters of three-point bending test (Figure 2, left and middle). The correlation is stronger for the flexural strength parameter and it applies for all types of provided comparisons (for both presented and discussed tests). It can be stated (with certain amount of caution) that influence on strength characteristics can be more easily predictable than on fracture work.

**Figure 3:**
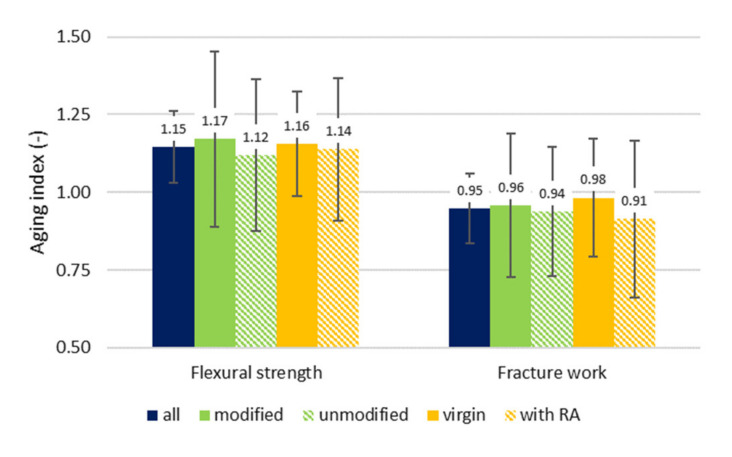
Average aging index—three-point bending test.

From overall evaluation (Figure 3), interesting conclusions can be deduced. The average aging index of flexural strength for “all” variants was 1.15. When the specimens were divided into groups according to the used binder, the mixtures with modified binder evinced worsen results, therefore the mixture were more influenced by the aging than asphalt mixtures with unmodified binder. It is unexpected, but it can indicate that polymers and additives used for modifications may not have always the long-term durability which we would wish for.

When the specimens were divided into groups according to RA content, again interesting phenomena occurred. In general, some of them were expected, but in several cases the resulting findings can be unexpected for some of the readers. The mixtures containing RA acted better after the aging than mixtures containing only virgin/raw materials. The already aged binder is usually less susceptible to further aging than new binder. The worst strain for the bituminous binder is undoubtedly manufacturing process in asphalt plant. The binder is exposed to high temperature and high pressures (“short-time aging”). After that, it is exposed to climate conditions and traffic loading (“long-term aging”). The maltenes fraction in binder transforms into asphaltenes due to oxidation processes [12]. This process can be partially rejuvenated by proper additives, but not fully. Additionally, it is still unclear how long this effect for different rejuvenators lasts. This means that the referential specimens of mixtures with RA include some of the aged binder from the beginning.

For aging indexes of fracture work, very opposite results have been identified. All the evaluated groups reached average aging index less than 1.0, so on average, the fracture work of aged test specimens is smaller than the virgin equivalent. Here the modified variants evinced better results, so the influence of aging on fracture work was smaller. On the other hand, the mixtures with RA showed worse results. The brittleness of aged bituminous binder becomes more evident—although the mixtures reached higher flexural strengths, the deformation ↔ fracture work was lower.

**Figure 4:**
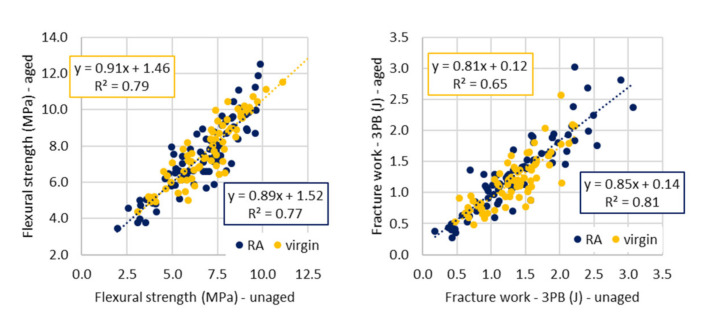
Comparison of aged and referential parameters for different groups of mixes—three-point bending test.

If correlation graphs are compared (Figure 4), it can be shown that the slope of the direction of linear regression (correlation) curve is similar for both of the parameters of “virgin” and “RA” groups. The direction ascends by a similar rate.

**Figure 5:**
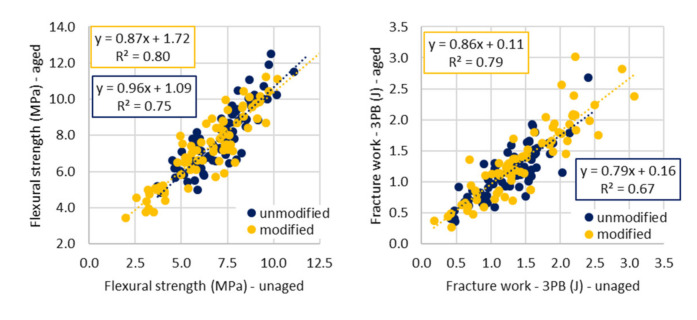
Comparison of aged and referential parameters for different groups of mixes—three-point bending test.

On the other hand, for “unmodified” and “modified” groups of test specimens (Figure 5), there are relevant differences. The slope of direction for the regression curve of “unmodified” groups is sharper, so the ascent is faster. For fracture work, notably, the opposite case occurred.

### 3.2. Semi-Circular Bending Test

SCB test in same cases of assessed asphalt mixtures confirms the conclusions from three-point bending test, but in several cases the results are diverse.

**Figure 6:**
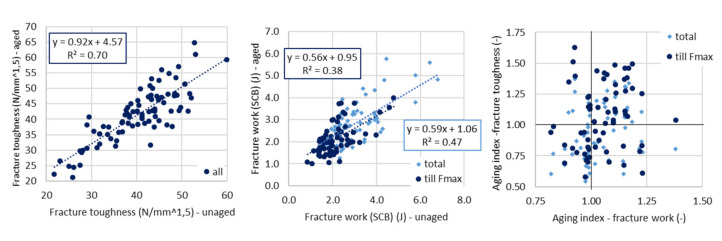
Comparison of aged and virgin parameters—semi-circular bending (SCB) test (**left** and **middle**) and comparison of aging indexes (**right**).

Again, the strength parameter (fracture toughness) correlates better than fracture work (Figure 6, right). For fracture toughness, the same conclusion applies as for flexural strength.

**Figure 7:**
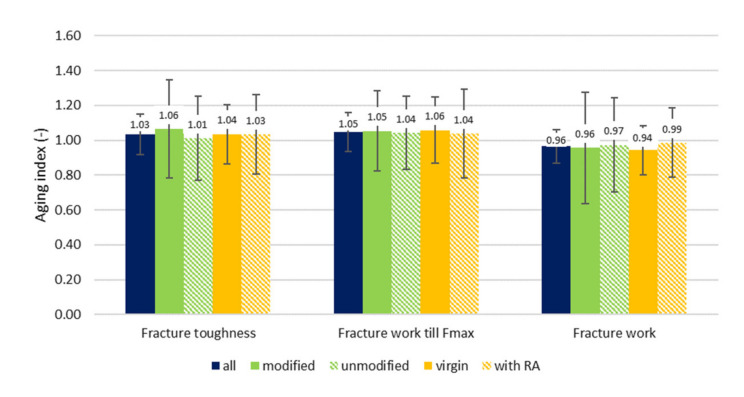
Average aging index—SCB test.

Even here it relates that the average aging index of fracture toughness is greater than 1.0, even though the values are more disarranged—approximately 40% of aging indexes are below 1.0. As it was stated above, the fracture toughness is influenced not only by the increased stiffness, but more likely by the increased brittleness, the notch at the bottom of the semi-circular specimens defines the weakest zone of the test specimen, which results in aging indexes above and below 1.0. The influence of modified bituminous binders is here again more evident (compare 1.06 vs. 1.01—Figure 7).

The fracture work till maximum force determined by the SCB test does not provide and confirm conclusions as were shown for 3PB test. For this parameter, the aging indexes are higher than 1.0, so on average the fracture work is higher for aged specimens. The trend in increase of aging indexes via selected groups is similar to one than fracture toughness shows—the “modified” and “virgin” groups are influenced more.

For total fracture work (including the unloading part of force-displacement diagram), again different conclusions have to be made if compared to 3PB test. All of the aging indexes are below zero—the brittleness of binder exceeds the toughness. “Unmodified” group and mixtures with RA are, in this case, less affected.

All the aging indexes and selected statistics are summarized in Table 1.

## 4. Discussion and Conclusions

The paper summarized some of the results of fracture parameters derived from SCB test and 3PB test after laboratory simulation of long-term aging. The aging was simulated by storing the specimens for 5 days in temperature of 85 °C with forced air circulation. After aging, the same procedure was performed as for unaged test specimens. The key objective of the paper was to compare the influence of aged binder from different perspective demonstrated on two low-temperature test method.

The results showed that strength properties (fracture toughness and flexural strength) derived from both tests on average increased by aging of bituminous binder. For three-point bending, the flexural strength increased in almost all cases. For fracture toughness, the increase was not that strong, but on average the fracture toughness increased as well. The flexural strength was mainly influenced by the stiffness modulus—the stiffer the mixtures are (to a certain level), the higher the flexural strength is. Fracture toughness is more likely influenced by the brittleness of the mixture at temperature of 0 °C than the stiffness itself.

For both tests, the results show that test specimens with modified bituminous binders (PMB and crumb rubber modified) were more influenced by the aging than the variants with unmodified (paving-grade) binders. The modified binders are mostly used for pavements on highways or heavy loaded roads. The results indicate that the stability of polymers or additives used for modifications may not have always the long-term durability which we would expect or wish for. Reclaimed asphalt (RA) content had a positive influence of lower aging indexes, so from the point of view of this assessed characteristic there is no need for worries in RA usage.

For fracture work (integral of force-displacement diagram), significantly different data were gained from both test methods. The three-point bending was performed on unnotched beams and SCB test was performed on notched semi-circular specimens. For 3PB, the fracture work decreased for aged specimens. The modified binders had positive influence on this characteristic (0.96 vs. 0.94) and the mixtures with RA reached better results (0.98 vs. 0.91).

For notched specimens (SCB test), the aging index in case of fracture work till maximum force (crack initiation) was greater than 1.0 and followed the same trend like fracture toughness, thus the asphalt mix groups denoted as “unmodified” and “RA” group showed better results, i.e., very opposite result than for 3PB test. For total fracture work (crack propagation), the impact of brittleness was more evident. All the aging indexes were below 1.0. Again the “unmodified” and “RA” group showed better results.

One of the additional results which can be taken from the data is that reclaimed asphalt content has no critically negative influence on the long-term durability of the mixtures. Based on the experimental results, it can be stated that no negative influence of reclaimed asphalt in asphalt mixtures after their aging was found in terms of the assessed fracture parameters; more likely the aging indexes were smaller for asphalt mixtures containing RA.

The results also show that modified binders are more susceptible to aging than unmodified binders. It has been shown that even though the modified binders have undoubtedly positive influence on most of the asphalt mix parameters, it is necessary to not forget about long term properties and focus on aging phenomena as well.

## Figures and Tables

**Table 1:** Aging index and related basic statistics.

	ALL					Modified					Unmodified				
	K_Ic_	W_Fmax_	W	R	W_3PB_	K_Ic_	W_Fmax_	W	R	W_3PB_	K_Ic_	W_Fmax_	W	R	W_3PB_
**Mean**	1.03	1.05	0.96	1.15	0.95	1.06	1.05	0.96	1.17	0.96	1.01	1.04	0.97	1.12	0.94
**Stand. error**	0.01	0.03	0.03	0.01	0.02	0.02	0.04	0.04	0.02	0.03	0.01	0.05	0.05	0.02	0.02
**St.deviation**	0.12	0.28	0.25	0.17	0.23	0.11	0.23	0.21	0.19	0.26	0.1	0.33	0.28	0.14	0.2
**Minimum**	0.74	0.48	0.49	0.81	0.51	0.82	0.61	0.58	0.81	0.53	0.82	0.48	0.49	0.83	0.51
**Maximum**	1.38	1.63	1.62	1.78	2.09	1.38	1.49	1.31	1.78	2.09	1.23	1.63	1.62	1.46	1.71
**Count**	88	71	70	147	145	43	34	34	76	73	42	37	36	71	73
	**Virgin**					**with RA**									
	K_Ic_	W_Fmax_	W	R	W_3PB_	K_Ic_	W_Fmax_	W	R	W_3PB_	Legend:				
**Mean**	1.04	1.06	0.94	1.16	0.98	1.03	1.04	0.99	1.14	0.91	K_Ic_ = fracture toughness				
**Stand. error**	0.02	0.05	0.04	0.02	0.03	0.02	0.05	0.04	0.02	0.02	W_Fmax_ = fracture work till F_max_				
**St. deviation**	0.11	0.28	0.25	0.19	0.25	0.12	0.29	0.24	0.14	0.2	W = total fracture work				
**Minimum**	0.82	0.58	0.49	0.81	0.53	0.74	0.48	0.58	0.82	0.51	R = flexural strength				
**Maximum**	1.38	1.49	1.35	1.78	2.09	1.31	1.63	1.62	1.46	1.71	W_3PB_ = fracture work				
**Count**	46	35	35	78	73	42	36	35	68	72					

## Data Availability

The data presented in this study are available on request from the corresponding author.
